# Hyperfibrinogenemia is associated with lymphatic as well as hematogenous metastasis and worse clinical outcome in T2 gastric cancer

**DOI:** 10.1186/1471-2407-6-147

**Published:** 2006-06-01

**Authors:** Hiroharu Yamashita, Joji Kitayama, Nobuko Kanno, Yutaka Yatomi, Hirokazu Nagawa

**Affiliations:** 1 Department of Surgical Oncology, The University of Tokyo, Graduate School of Medicine, Tokyo, Japan; 2 Department of Laboratory Medicine, The University of Tokyo, Graduate School of Medicine, Tokyo, Japan

## Abstract

**Background:**

Abnormal hemostasis in cancer patients has previously been described, however the correlation between the plasma fibrinogen level and cancer metastasis and prognosis has not been reported in a large-scale clinical study.

**Methods:**

Preoperative plasma fibrinogen levels were retrospectively examined in 405 patients who underwent surgery for advanced gastric cancer. The association of fibrinogen levels with clinical/pathological findings and clinical outcome was evaluated.

**Results:**

There was a positive correlation between plasma fibrinogen levels and the depth of invasion (p < 0.05). Hyperfibrinogenemia (>310 mg/dl) was independently associated with lymph node (Odds Ratio; 2.342, P = 0.0032) and liver (Odds Ratio; 2.933, P = 0.0147) metastasis, not with peritoneal metastasis in this series. Patients with hyperfibrinogenemia showed worse clinical outcome in T2 gastric cancer, however, there was no correlation of plasma fibrinogen level with prognosis in T3/T4 gastric cancer.

**Conclusion:**

Our results might support the idea that hyperfibrinogenemia can augment lymphatic and hematogeneous metastasis of advanced gastric cancer, which is major determinant of the prognosis in T2 gastric cancer. Therefore, in the situation without peritoneal involvement, hyperfibrinogenemia is a useful biomarker to predict the possible metastasis and worse clinical outcome in T2 gastric cancer.

## Background

An increased frequency of thrombosis in patients with gastrointestinal cancer was first documented in 1865 [1]. Since then, a number of studies have focused on the relationship between various cancers and hemostatic factors. Abnormalities in blood coagulation are detectable in patients with malignancy, including thrombocytosis and elevated markers of coagulation activation such as fragment 1+2, thrombin-antithrombin III complexes (TAT), fibrinopeptide A (FPA), and D-Dimer [2-4]. More recently, a correlation between these factors and the prognosis of malignancies was documented. Thrombocytosis is thought to be associated with poor prognosis in gastric cancer [5], as well as esophageal cancer [6], lung cancer [7], colon cancer [7], renal cell carcinoma [8], and gynecological malignancies [9], D-Dimer is also reported to be associated with poor prognosis in patients with lung cancer [10,11] and colorectal cancer [12,13] as well as being a good predictor of survival and disease progression.

Fibrinogen, an essential hemostatic factor, is converted to fibrin (a final product of the hemostatic pathway) by activated thrombin. Dvorak suggested that elevated fibrinogen concentrations were frequently observed in cancer patients with malignant disease [14]. Moreover, in gastric cancer, preoperative plasma fibrinogen levels correlate with extent of tumor [15]. In our previous study, we found that preoperative plasma fibrinogen level was a useful predictor for lymph node metastasis in patients with gastric cancer [16]. Tumor marker carcinoembryonic antigen (CEA) as well as inflammatory biomarker C-reactive protein (CRP) did not show the independent association with lymph node metastasis, suggesting that hyperfibrinogenemia was not a simple result of cancer progression and might support the metastatic process by providing beneficial microenvironment around the tumor. This idea can be partially supported by recent studies in fibrinogen-deficient mice that revealed hematogeneous and lymphatic metastases were greatly reduced, indicating the positive roles of fibrinogen in the metastatic progression of cancer [17-19]. Taken together with our previous results, we hypothesized that fibrinogen might augment metastasis in human as well, and a higher level of plasma fibrinogen might be a good clinical marker of metastatic disease and worse clinical outcome. Based on this assumption, we focused on the possible impact of preoperative plasma fibrinogen level on gastric cancer prognosis. We retrospectively examined the preoperative plasma fibrinogen level of patients with advanced gastric cancer who underwent gastrectomy, and evaluated an association of these findings with clinical and pathological factors as well as clinical outcome.

## Methods

A total of 442 patients with advanced gastric cancer underwent gastrectomy between January 1985 and December 1999 at the First Department of Surgery, University of Tokyo Hospital, Tokyo. Oral consent was obtained from each patient for blood test. Among them, plasma fibrinogen levels were evaluated before surgery in 424 patients. Because the plasma fibrinogen level is critically affected by the presence of inflammation, liver cirrhosis and chronic renal failure, five patients with apparent acute inflammatory disease (two with acute cholecystitis, three with acute peritonitis due to stomach perforation), 10 patients with liver cirrhosis and four patients with chronic renal failure were excluded from the study to minimize confounding factors. The remaining 405 patients were enrolled in this study. We also evaluated fibrinogen levels in 387 patients with early gastric cancer and 124 patients with non-inflammatory benign disease who underwent surgery during the same period. Our protocol was approved by the ethics committee of faculty of medicine, the University of Tokyo.

In this study we referred to the classifications established by the Japanese Research Society for Gastric Cancer [20] which define T1 as a lesion confined to the mucosal or submucosal layer; T2 as a tumor invading the proper muscle layer or subserosa; T3 as a tumor penetrating serosal without invasion of adjacent structures; and T4 as a tumor invading adjacent structures. Histological classifications were defined as follows: differentiated carcinoma – well and moderately differentiated tubular adenocarcinoma and papillary adenocarcinoma; undifferentiated carcinoma – poorly differentiated adenocarcinoma, signet ring cell carcinoma and mucinous carcinoma.

The preoperative plasma fibrinogen level was measured from early morning samples taken before breakfast five to ten days before surgery. It had been determined by Clauss clotting method using DADE Thrombin Reagent™ and Coagrex-700™ automated coagulometer (both from Sysmex, Kobe, Japan). The reference range of plasma fibrinogen level was defined as between 210 and 310 mg/dl according to the mean value ± 2 SD of healthy volunteers; plasma fibrinogen levels above 310 mg/dl were defined as hyperfibrinogenemia tentatively in this study.

Statistical analysis was carried out using JMP 5.1 (SAS Institute, Cary, NC). Plasma fibrinogen levels were compared with one-way ANOVA followed by the SNK test. The association of fibrinogen levels with clinicopathological factors was assessed with Fisher's exact test. A multivariate stepwise logistic regression analysis was performed to identify independent variables that were correlated with hematogenous and lymphatic metastasis. The Kaplan-Meier method was used to estimate the distribution of survival curve, and log-rank test was used to compare the distributions between the groups with or without hyperfibrinogenemia. P < 0.05 was considered significant for all statistical analyses.

## Results

### The association of plasma fibrinogen level with the depth of invasion and metastatic disease in gastric cancer

**Figure 1:**
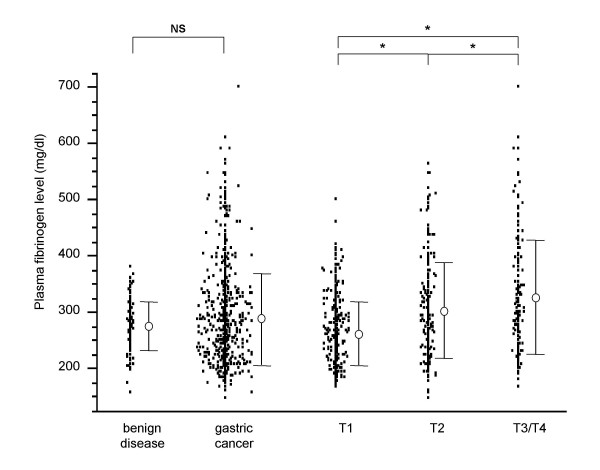
Plasma fibrinogen level of 124 patients with benign disease and 792 patients with gastric cancer. Note that there is no difference in fibrinogen levels between benign and cancer patients, although it gradually increases with increasing depth of cancer invasion (one way ANOVA with SNK test; p < 0.0001). Values are the mean (standard deviation) levels for individual patients. *The mean values of plasma fibrinogen were significantly different between the groups of T1 and T2 (P < 0.01), between the groups of T1 and T3/T4 (P < 0.01), and between the groups of T2 and T3/T4 (p < 0.01).

The mean ± standard deviation (SD) plasma fibrinogen level in the 792 patients studied was 289.5 ± 81.3 mg/dl, which was not statistically different from patients with benign disease (278.1 ± 43.5) (Figure 1). However, when patients were classified into 3 groups according to the T classification of gastric cancer, patients with T2 and T3/T4 cancer showed significantly higher fibrinogen levels than those with benign disease or those with T1 cancer (T2 304.3 ± 85.0, p < 0.01; T3/T4 327.8 ± 101.2, p < 0.01). Interestingly, an increase in the plasma fibrinogen level correlated with an increase in the depth of invasion, showing a statistically significant difference among patients when evaluated by ANOVA (p < 0.0001). The fibrinogen levels in patients with localized disease, lymph node metastases, liver metastasis and peritoneal metastasis were summarized in Table 1. In patients with localized disease, preoperative fibrinogen level (mean value; 266.6) was significantly lower than that in patients with lymph node metastasis (320.5; P < 0.0001), liver metastasis (356.6; P < 0.0001), peritoneal metastasis (345.0; P < 0.0001).

### The association of hyperfibrinogenemia with lymph node, liver, and peritoneal metastasis in advanced gastric cancer

Of 405 patients with advanced cancer, lymph node metastases in 301 patients, liver metastases in 28, and peritoneal metastases in 41 were identified. Univariate analysis revealed that lymph node metastases were significantly associated with many pathologic factors such as size of the tumor, serosal invasion, lymphatic and venous involvement as well as plasma fibrinogen level (Table 2). Multivariate analysis indicated that hyperfibrinogenemia showed an independent association with lymph node metastases with an odds ratio of 2.342 (p < 0.01) (Table 3). Subsequently, we focused on distant hematogeneous and peritoneal metastases. In individuals with advanced gastric cancer, hyperfibrinogenemia showed a positive association with liver metastasis (p < 0.01), not with peritoneal dissemination, by univariate analysis (Table 2). Multivariate analysis showed that hyperfibrinogenemia, in addition to differentiated histology and venous involvement, remained independently associated with liver metastasis with an odds ratio of 2.933 (p < 0.05) (Table 3).

### The clinical outcome in T2 and T3/T4 gastric cancer with or without hyperfibrinogenemia

**Figure 2:**
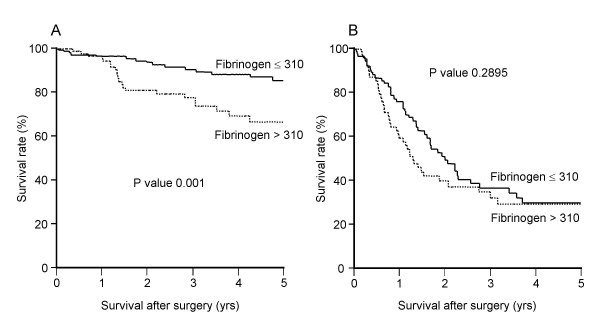
Overall survival after surgery for 240 patients with T2 gastric cancer (A) and 165 patients with T3/T4 gastric cancer (B) according to the existence of hyperfibrinogenemia. The group with hyperfibrinogenemia (>310 mg/dl) showed a significantly lower survival rate than another group (≤310 mg/dl) in T2 cancer. There was no statistically significant survival difference in T3/T4 cancer according to the plasma fibrinogen level.

In T2 gastric cancer, patients without hyperfibrinogenemia showed an extremely good outcome even in the population with advanced cancer, and the survival rate was significantly lower for patients with hyperfibrinogenemia (P = 0.001; log-rank test) (Figure 2A). In marked contrast, hyperfibrinogenemia did not show any correlation with 5-year survival rate in T3/T4 gastric cancer, i.e. cancer invading beyond the serosal (Figure 2B).

## Discussion

A link between hyperfibrinogenemia and cardiovascular diseases such as coronary heart disease, stroke and peripheral vascular disease was previously revealed [21]. Similarly, a recent study by Preston and colleagues indicates that fibrinogen production is upregulated in patients with pancreatic adenocarcinoma, although the tumor stage was not determined [22]. This suggests a positive role for fibrinogen in the progression of malignant diseases. In the present study we found that fibrinogen levels in patients with gastric cancer did not show statistically significant difference from those in individuals with benign diseases. However, the plasma fibrinogen level gradually increased with increasing depth of tumor, and the fibrinogen levels of patients with advanced gastric cancer were significantly higher than patients with benign diseases or early tumors. Moreover, the fibrinogen levels were also significantly higher in patients with metastatic disease, which essentially supports the previous study of another group [15]. Hyperfibrinogenemia is a clinically relevant event in advanced stage and our results strongly suggest that fibrinogen is involved in the progression of gastric cancer during the latter phase of the disease.

Recent studies in fibrinogen deficient mice provide clear evidence that fibrinogen plays a crucial role in hematogenous and lymphatic metastasis of cancer cells [18]. They showed that fibrinogen (Aα-chain)-deficient mice with intravenously transferred Lewis lung carcinoma (LLC) or B16 melanoma had a significantly reduced incidence of lung metastasis compared with wild type mice [17]. Also, the number of metastases in regional lymph nodes and the lungs of these transgenic mice was markedly reduced when LLC was subcutaneously inoculated [18]. These results raised the possibility that hyperfibrinogenemia might function to enhance metastasis formation as compared to the low fibrinogen level. As a fact, hyperfibrinogenemia showed an independent association with lymph node metastasis in human gastric cancer [16]. Interestingly, there was no remarkable difference in the growth of subcutaneously transplanted tumors between fibrinogen-deficient and wild type mice [18], indicating that fibrinogen plays a major role in the development of metastases but not in the growth of the primary tumor. This is exactly consistent with our human study in which patients with early gastric cancer had a similar plasma fibrinogen level to benign subjects.

In our study, we found that plasma fibrinogen level was associated with worse prognosis in T2 gastric cancer as well as lymphatic and hematogenous metastasis. The impact of hyperfibrinogenemia, however, was not found in T3/T4 gastric cancer. T3/T4 gastric cancers are serosal-infiltrating tumors, which enhances the potential to have occult metastatic foci on the peritoneum and/or form the overt peritoneal metastasis. As a fact, peritoneum is the major site of recurrence in T3/T4 gastric cancers [23-25], and the prognosis of T3/T4 cancers mainly determined by this type of recurrence. In contrast, T2 cancer has a less frequency of peritoneal metastasis as compared with T3/T4 cancer and liver or lymph node relapse seems to be major determinant of clinical outcome in T2 cancer. In our study, however, we found no association of hyperfibrinogenemia with peritoneal metastasis, although hyperfibrinogenemia was an independently associated factor with lymph node and liver metastasis. We speculate that peritoneal metastasis has a strong impact on the clinical outcome in T3/T4 gastric cancer and therefore plasma fibrinogen level was not a prognostic marker in this population. These results suggested that some patients with hyperfibrinogenemia could not be cured by surgical modalities in T2 gastric cancer and chemotherapy might be required for the improvement of survival in this population. The clinical benefit of neoadjuvant and/or adjuvant chemotherapy has not been clearly determined in T2 gastric cancer. From our data, we can conclude that patients with hyperfibrinogenemia might be good candidates for neoadjuvant and/or adjuvant chemotherapy in T2 gastric cancer. Patients with T3/T4 gastric cancer will be candidates regardless of the plasma fibrinogen level.

Fibrinogen may enhance metastasis through several possible mechanisms. Firstly, the soluble form of fibrinogen could serve as the bridging molecule between tumor cells and host cells. Fibrinogen is a dimeric molecule with multiple integrin or non-integrin binding motifs, and malignant cells often express high levels of fibrinogen receptors, such as α5β1, αvβ3 integrins or ICAM-1 molecule. If fibrinogen were to bind to ICAM-1 on endothelial cells, it might promote stable adhesion of tumor cells to the endothelium of target organs. In addition, tumor cells and platelets can form large aggregates through the binding of fibrinogen, because platelet αIIbβ3 integrin receptors have a high affinity for fibrinogen. These aggregates effectively form microemboli in target organs, which can protect tumor cells from the innate immune system [26,27]. A study by Palumbo and colleagues found that the number of tumor cells located in the lung was markedly decreased in fibrinogen deficient mice after 4–24 hours of tumor inoculation, but at no earlier time points. This suggests that fibrinogen is essential for the sustained adherence of tumor cells to the endothelia of target organs [17].

Recent studies have shown that thrombin might also be an important modulator of cancer metastasis *in vivo*. During the conversion of prothrombin to thrombin by prothrombinase, the polypeptide prothrombin fragment 1+2 (F1+2) is released. The plasma F1+2 level is therefore a useful marker of thrombin generation. A number of studies have shown that F1+2 plasma levels are elevated in patients with specific types of malignancies [4,28,29] but not in other types [30-32]. Rahr and coworkers reported no difference in plasma F1+2 levels between patients with and without gastric cancer [32]. In the present study, prothrombin time (PT) was examined in each patient and no significant association with the presence of cancer, metastasis, tumor stage or plasma fibrinogen level was found (data not shown). Moreover, Kerlin and co-workers recently demonstrated that elevated fibrinogen levels in hyperfibrinogenemia transgenic mice with suppressed thrombin activity do not alter the incidence or extent of thrombus formation [33]. It therefore seems unlikely that high fibrinogen levels are associated with systemic thrombin activation and subsequent enhancement of fibrin formation in gastric cancer.

Although there is sufficient evidence to suggest that elevated fibrinogen levels might aid the development of metastatic lesions, we cannot ignore the possibility that this elevation is simply the result of the tumor mass spreading. Fibrinogen, which is one of the major acute phase proteins produced by the liver, is greatly enhanced in response to infection or other inflammatory disorders. Indeed, the fibrinogen level of all of the patients with preoperative acute inflammatory disorders was high. Inflammatory proteins, such as IL-6 or CRP, are reported to be higher in cancer patients compared to non-cancer patients. In our previous study, we found that CRP level did not show an independent association with lymphatic metastasis although plasma fibrinogen level did [16], suggesting that hyperfibrinogenemia, different from high serum CRP, may not be a simple by-product of inflammatory response caused by tumor progression but might have some etiologic relevance for tumor metastasis and accordingly relationship with clinical outcome in gastric cancer. In this retrospective study as well as our previous study, however, we could not show the exact causal relationship between hyperfibrinogenemia and cancer metastasis.

## Conclusion

Metastasis is the unequivocal hallmark of cancer. The acquisition of metastatic ability leads to clinically incurable disease and resultingly worse clinical outcome for most cancer cell types. Our data, together with the results of basic experiments in previous studies, raises the idea that hyperfibrinogenemia causally imparts the hematogenous and lymphatic metastatic progression in patients with advanced gastric cancer, and is not simply the result of tumor progression. It also has a prognostic value in T2 gastric cancer, which might suggest hyperfibrinogenemia has the potential to be prognostic marker in other cancer type with less frequent peritoneal metastasis. Routinely examined plasma fibrinogen level is not only the factor to assess the perioperative hemorrhagic risk but also a useful biomarker to predict the possible metastasis and worse prognosis in T2 gastric cancer.

## Abbreviations

thrombin-antithrombin III complexes: TAT, fibrinopeptide A: FPA, carcinoembryonic antigen: CEA, C-reactive protein: CRP, Lewis lung carcinoma: LLC, prothrombin fragment 1+2: F1+2, prothrombin time: PT.

## Competing interests

The author(s) declare that they have no competing interests.

## Authors' contributions

**HY **have been involved in preparing the manuscript for submission and made substantial contributions to conception and design, acquisition of data, and analyses and interpretation of data;

**JK **contributed to analyses and interpretation of data and their interpretation and was involved in revising the manuscript for important intellectual content;

**NK **and **YY **have been involved in plasma fibrinogen measurement and data analyses;

**HN **participated in the design of the study and was involved in discussion of the results.

## Pre-publication history

The pre-publication history for this paper can be accessed here:



## Figures and Tables

**Table 1:** Preoperative plasma fibrinogen level of the patients with or without metastases.

	number	plasma fibrinogen level (mg/dl)	s.d.
localized disease	456	266.6	62.8
lymph node metastasis	336	320.5	92.7
liver metastasis	28	356.6	94.0
peritoneal metastasis	41	345.0	92.9

s.d; standard deviation

**Table 2:** Relationship between clinicopathological factors and metastasis in advanced gastric cancer

		**lymph node metastasis**			**liver metastasis**			**peritoneal metastasis**		
**variables**	**total**	**negative**	**positive**	**P value**	**negative**	**positive**	**P value**	**negative**	**positive**	**P value**
age (years)										
≤59	192	56	136		183	9		170	22	
≥60	213	48	165	0.1394	194	19	0.1168	194	19	0.4142
gender										
male	284	76	208		264	20		258	26	
female	121	28	93	0.5345	113	8	>0.9999	106	15	0.3684
tumor location										
upper	124	31	93		117	7		107	17	
middle	152	40	112		143	9		134	18	
lower	129	33	96	0.969	117	12	0.4303	123	6	0.0389
differentiation										
differentiated	180	46	134		162	18		165	15	
undifferentiated	225	58	167	>0.9999	215	10	0.0314	199	26	0.3225
lymphatic involvement										
negative	125	68	57		119	6		119	6	
positive	280	36	244	<0.0001	258	22	0.2974	245	35	0.0196
venous involvement										
negative	195	60	135		190	5		180	15	
positive	210	44	166	0.0303	187	23	0.0008	184	26	0.1386
serosal invasion										
negative	240	91	149		226	14		235	5	
positive	165	13	152	<0.0001	151	14	0.3236	129	36	<0.0001
tumor size (cm)										
≤6	215	80	135		202	13		206	9	
>6	190	24	166	<0.0001	175	15	0.5571	158	32	<0.0001
Plasma fibrinogen (mg/dl)										
≤310	234	76	158		226	8		216	18	
≥311	171	28	143	0.0002	151	20	0.0014	148	23	0.067

**Table 3:** Relationship between clinicopathological factors and metastasis in advanced gastric cancer (multivariate analysis)

**lymph node metastasis**				
	odds ratio	95% CI		P value
serosal invasion	3.983	1.991	7.970	<0.0001
tumor size	1.965	1.088	3.546	0.0251
lymphatic involvement	5.780	3.333	10.000	<0.0001
venous involvement	0.746	0.426	1.308	0.3067
hyperfibrinogenemia	2.342	1.330	3.546	0.0032

**liver metastasis**				
	odds ratio	95% CI		P value

differentiated type	2.439	1.070	5.556	0.0341
venous involvement	4.463	1.631	12.214	0.0036
hyperfibrinogenemia	2.933	1.236	6.944	0.0147
